# Genotypic Analysis of *Candida tropicalis* Clinical Isolates From Korea via Multilocus Sequence Typing

**DOI:** 10.1002/mbo3.70024

**Published:** 2025-06-29

**Authors:** Hye‐won Park, Lia Kim, Yoon‐Sung Choi, Jinyoung Bae, Min‐Ho Yeo, Eun Ju Lee, Jiyoung Lee, Kwangmin Park, Dong Geon Lee, Min Park, Sunghyun Kim, Jungho Kim

**Affiliations:** ^1^ Department of Clinical Laboratory Science, College of Health Sciences Catholic University of Pusan Busan Republic of Korea; ^2^ Department of Biomedical Laboratory Science, College of Health Sciences Yonsei University Wonju Republic of Korea; ^3^ Department of Thoracic and Cardiovascular Surgery, Inje University Haeundae Paik Hospital Inje University College of Medicine Busan Republic of Korea; ^4^ Korea Mycobacterium Resource Center (KMRC), Department of Research and Development The Korean Institute of Tuberculosis, Osong Republic of Korea; ^5^ Department of Research & Development DreamDX Inc. Busan Republic of Korea; ^6^ Next‐Generation Industrial Field‐Based Specialist Program for Molecular Diagnostics, Brain Busan 21 Plus Project, Graduate School Catholic University of Pusan Busan Republic of Korea; ^7^ Department of Biomedical Laboratory Science Masan University Changwon Republic of Korea

**Keywords:** *Candidia tropicalis*, genetic diversity, multilocus sequence typing, non‐albicans Candida species

## Abstract

*Candida tropicalis* is a clinically significant non‐albicans species that has shown increasing azole resistance globally. Despite its growing clinical importance, genotypic and epidemiological data from East Asia, particularly Korea, remain limited. This study aimed to expand the global understanding of *C. tropicalis* population structure by genotyping clinical isolates from Korea and identifying novel sequence types. Thirty‐four clinical isolates collected from tertiary hospitals across Korea between 2012 and 2019 were analyzed using multilocus sequence typing (MLST) based on six housekeeping genes (ICL1, MDR1, SAPT2, SAPT4, XYR1, and ZWF1). A total of 21 diploid sequence types (DSTs) were identified, including three novel DSTs (1418, 1419, and 1420) not previously recorded in the MLST central database. Notably, DST844 (CTR‐19 isolate) exhibited elevated minimum inhibitory concentration (MIC) to fluconazole, suggesting a potential novel resistance phenotype. Phylogenetic and goeBURST analyses revealed 11 distinct clonal complexes (CCs), some of which showed similarity to Chinese and Brazilian lineages, indicating cross‐regional evolutionary links. These findings enhance the global MLST data set, provide new insights into the molecular epidemiology of *C. tropicalis* in Korea, and highlight the need for continued antifungal resistance surveillance, particularly for emerging DSTs.

## Graphical abstract

Multilocus sequence typing of 34 *Candida tropicalis* clinical isolates from Korea revealed three novel sequence types and evidence of genetic recombination. These findings highlight the ongoing genetic diversification and underscore the need for integrated genomic surveillance of emerging antifungal‐resistant strains.

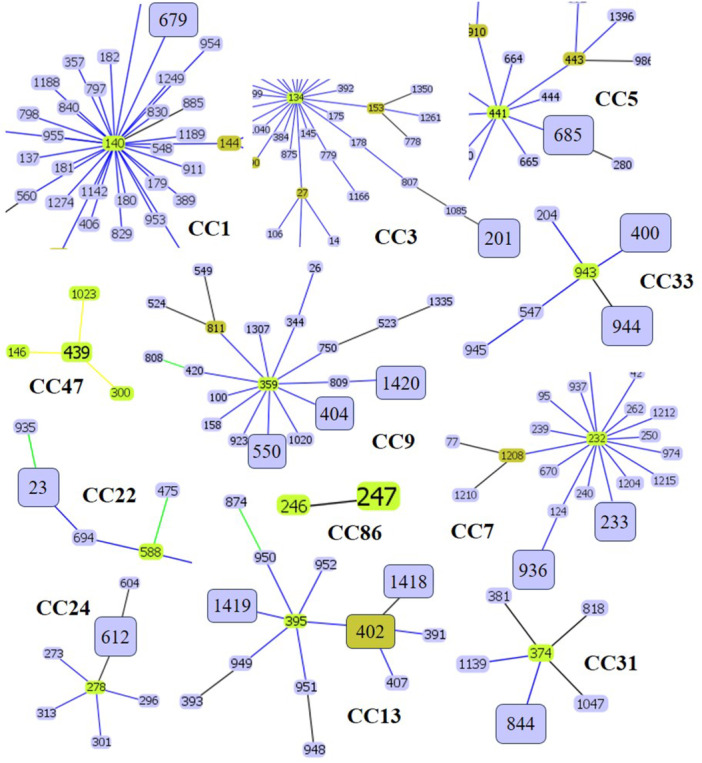

## 1 Introduction

Severe fungal infections affect approximately 300 million individuals of all age groups annually, resulting in over 1.5 million deaths worldwide (Brown et al. 2012; Del Poeta 2016; Sims et al. 2005). Among these infections, Candida species are major opportunistic pathogens, particularly in immunocompromised patients and those with prolonged hospital stays (Quindós 2014; Wang, Li et al. 2020). These fungi are responsible for both superficial and invasive infections and are increasingly recognized as significant causes of hospital‐acquired infections, posing growing public health challenges (Sims et al. 2005; Wang, Li et al. 2020).

While *Candida albicans* remains the most commonly isolated species, non‐albicans Candida (NAC) species such as *Candida tropicalis* are gaining prominence due to their increasing incidence and antifungal resistance (Falagas et al. 2010). In particular, *C. tropicalis* has emerged as a leading cause of candidemia in patients with hematologic malignancies and those in intensive care units. Its high mortality rate and frequent resistance to azole antifungals highlight the need for enhanced surveillance and characterization (Tan et al. 2015; Wu et al. 2014, 2017). Although global surveillance studies have documented *C. tropicalis* as prevalent in Latin America and the Asia‐Pacific region, recent data also show a rise in Mediterranean and African countries (Tan et al. 2015; Wu et al. 2017; Arastehfar, Hilmioğlu‐Polat et al. 2020; Kumar et al. 2014; Megri et al. 2020; Osman et al. 2020; Scordino et al. 2018). In Korea, the Korean National Healthcare‐associated Infections Surveillance (KONIS) system reports *C. tropicalis* as the second most prevalent Candida species among hospitalized patients, accounting for 22% of isolates, following *C. albicans* at 38% (Kim et al. 2020). However, molecular epidemiological data specific to *C. tropicalis* in Korea remain limited, creating a knowledge gap in understanding its genotypic diversity, resistance patterns, and transmission dynamics within this region.

Given the diploid nature of *C. tropicalis* and the rising concern over antifungal resistance, multilocus sequence typing (MLST) offers a robust and standardized method for elucidating population structure, tracking clonal expansion, and comparing strains across regions (Butler et al. 2009; Zuza‐Alves et al. 2017). Most genotypic studies to date have focused on *C. albicans*, leaving the genetic landscape of *C. tropicalis* underexplored, especially in Korea (Wu et al. 2019). Therefore, this study aimed to perform MLST‐based genotyping of *C. tropicalis* clinical isolates collected from Korean hospitals to expand the global allele database, identify novel sequence types, and explore their epidemiological and clinical significance (Boonsilp et al. 2021).

In this study, we conducted a retrospective investigation of 34 *Candida tropicalis* clinical isolates collected from multiple sites in Korea, with the aim of enriching the central MLST allele database and examining genetic diversity, evolutionary trends, and molecular epidemiology across globally distributed *C. tropicalis* populations.

## 2 Materials and Methods

### 2.1 Clinical Isolates and Antifungal Drug Susceptibility

From 2012 to 2019, 34 clinical *C. tropicalis* isolates were obtained from the Korean Culture Collection of Medical Fungi (KCMF). These isolates were obtained from tertiary general hospitals in Korea, including the Asan Medical Center in Seoul, Wonju Severance Christian Hospital in Wonju, and Chungbuk National University Hospital in Cheongju. *C. tropicalis* clinical isolates were obtained from blood, closed pus, ascitic fluid, and other clinical specimens (Supporting Information S1: Table S1).

Antimicrobial testing was performed using the minimum inhibitory concentration (MIC) values based on the MIC analytical sensitivity criteria for each isolate, following the guidelines established by the Clinical and Laboratory Standards Institute (Supporting Information S1: Table S2).

Clinical isolates were preserved in Sabouraud dextrose broth with 40% glycerol at –80°C until use. For DNA extraction, the clinical isolates were inoculated into Sabouraud dextrose agar and incubated at 37°C. Pure yeast cultures were obtained by inoculating a single colony into SDB, after which the single colony was used for further assays.

### 2.2 Genomic DNA Extraction

Total genomic DNA (gDNA) of the isolates was extracted using the I‐genomic BYF DNA Extraction Mini Kit (iNtRON Inc., Seongnam, Korea), following the manufacturer's instructions. A single colony was cultured in a liquid medium, and the cultured yeast sample was centrifuged to form a pellet. After adding 200 µL of MYP buffer and 2 µL of β‐mercaptoethanol, the pellet was incubated at 37°C for 15 min. The supernatant was removed via centrifugation. The sample was lysed with 100 μL of MP buffer and 2 μL of lyase solution (4.2 units/μL), incubated at 37°C for 15 min, and mixed every 3 min to ensure complete dissolution. After centrifugation, the supernatant was discarded. The cell pellet was completely dissolved by adding 200 µL of MG buffer, 20 µL of proteinase K solution (20 mg/mL), and 5 µL of RNase A solution (10 mg/mL). For high‐purity DNA extraction, the transparent lysate was analyzed after incubation at 65°C for 30 min. Next, 250 µL of MB buffer and 250 µL of 80% ethanol were added, and the lysate was transferred to a column in a 2.0‐mL collection tube, centrifuged, and the lysate was washed. Finally, EB buffer was added to elute the DNA.

Concentration and purity of gDNA were assessed using the Nanodrop 2000 spectrophotometer (Thermo Scientific, Wilmington, DE, USA) by measuring the optical density at 260/280 nm. The extracted gDNA was stored at 4°C until use.

### 2.3 Molecular Identification of *C. tropicalis* Clinical Isolates

In this study, an internal transcribed spacer (ITS), commonly used for yeast species identification, was used to identify the *Candida* species, as previously described (Scordino et al. 2018). ITS1 and ITS4 primers were used to amplify the *ITS* genes (Supporting Information S1: Table S3). The extracted gDNA was subjected to PCR using PrimeTaq Primix (Genet Bio Inc., Daejeon, Korea). PCR conditions were as follows: initial denaturation at 94°C for 60 s, followed by 40 cycles of denaturation at 94°C for 30 s, annealing at 57°C for 30 s, and extension at 72°C for 45 s, with a final extension at 72°C for 420 s. PCR‐amplified DNA fragments were visualized via 1.5% gel electrophoresis to confirm a single band of the desired product and sequenced to determine a partial base sequence of 560 bp.

### 2.4 MLST Analysis

MLST was conducted using six housekeeping genes: *ICL1*, *MDR1*, *SAPT2*, *SAPT4*, *XYR1*, and *ZWF1a (*Tavanti et al. 2005 *)*. Each gene underwent separate PCR amplification with specific primers (Supporting Information S1: Table S4) PCR was carried out using a mixture of 10 μL Prime Taq Primix (Genet Bio Inc.), 3 μL of gDNA template, 5 μL of distilled ultrapure water, and 1 μL of each primer (10 pmol/μL). The amplification conditions included an initial denaturation at 94°C for 7 min, followed by 40 cycles of denaturation at 94°C for 60 s, and annealing at the primer Tm (51°C–58°C) for 60 s. This was followed by an initial extension at 72°C for 60 s and final extension at 72°C for 10 min, with the reaction held at 4°C. The amplified product was confirmed by visualizing a single band of the desired size via 1.5% gel electrophoresis. PCR products that produced the band at the expected position were sequenced by Macrogen Inc. (Seoul, Korea).

Acquired sequence data were visually inspected using the MEGA 11 software (http://www.megasoftware.net) before performing MLST. This process facilitated the identification and correction of errors in genetic interpretation. Sequence data for each locus were compared with the sequences on the PubMLST database (https://pubmlst.org/ctropicalis) to assign allele numbers. Newly identified diploid sequence types (DSTs) were registered using a database curator after careful review. To register new DSTs from this study, gene and sample information were compiled and submitted to the curator. The registration was confirmed within a few days of evaluation.

### 2.5 Phylogenetic Analysis and Global Population Structure of *C. tropicalis*

Phylogenetic relationships among gene sequences were evaluated through cluster analysis using the unweighted pair group method with arithmetic mean (UPGMA) and p‐distance metrics in MEGA 7 (http://www.megasoftware.net/). Node support within the UPGMA dendrogram was tested via bootstrap analysis with 1000 replicates, and bootstrap values of 70% or higher were regarded as statistically significant.

The goeBURST algorithm with the PHILOVIZ 2.0 software (http://www.phyloviz.net/) was used to identify the clonal complexes (CCs) among the isolated loci (http://eburst.mlst.net). This algorithm determines associations in the same CC via single‐locus variant analysis. If five of the six genes used in MLST were identical, they were considered to belong to the same CC. The groups were designated with Arabic numerals as groups 1, 2, and 3. Additionally, a minimum spanning tree was constructed from the concatenated DST sequences using the GrapeTree software (https://achtman-lab.github.io/GrapeTree).

### 2.6 Pairwise Homoplasy Index Test

Using MLST data, potential recombination events among *Candida topicalis* isolates were investigated by performing a Pairwise Homoplasy Index (PHI) test with SplitsTree version 4.19.2. The Split Decomposition method was applied in the PHI test to visualize conflicting phylogenetic signals indicative of recombination.

## 3 Results

### 3.1 Characteristics of the Study Participants

In this study, a total of 34 *Candida tropicalis* isolates were collected from various clinical specimens, including blood (24/34, 70.6%), closed pus (3/34, 8.8%), ascitic fluid (2/34, 5.9%), bile (1/34, 2.9%), and other sources (4/34, 11.8%). The demographic and clinical profiles of the patients are summarized in Supporting Information S1: Table S1. Patient ages ranged from 32 to 98 years, with a mean age of 68.47 ± 14.19 years. Males comprised 64.7% of the cohort (22 out of 34 patients). The most frequently observed underlying conditions were cancer (16/34, 47.0%) and pneumonia (5/34, 14.7%).

### 3.2 *C. tropicalis* Strain Differentiation via MLST

DNA sequences from six housekeeping genes—ICL1, MDR1, SAPT2, SAPT4, XYR1, and ZWF1a—were analyzed in 34 clinical *Candida tropicalis* isolates (Table 1). Using the MLST scheme, fragments ranging from 479 to 737 base pairs within the coding regions were sequenced, generating a total alignment of 3,667 nucleotides per isolate. In total, 52 unique alleles were identified across the six loci. Among them, the XYR1 locus exhibited the highest diversity with 14 alleles, followed by MDR1 with 11, SAPT4 with 9, ZWF1a with 8, and both SAPT2 and ICL1 with 5 alleles each.

**Table 1:** List of diploid sequence types (DSTs) identified via multilocus sequence typing (MLST).

Sample	MLST loci						DST
	ICL1	MDR1	SAPT2	SAPT4	XYR1	ZWF1	
CT‐1	1	3	3	17	6	3	679
CT‐2	1	24	3	7	24	7	233
CT‐3	1	22	12	17	2	7	685
CT‐4	1	7	4	7	85	7	612
CT‐5	1	46	12	76	60	9	708
CT‐6	10	58	4	8	3	3	247
CT‐7	1	19	2	14	100	3	404
CT‐8	3	7	4	6	75	10	201
CT‐9	5	7	3	23	18	7	944
CT‐10	1	101	4	56	23	37	1418
CT‐11	37	100	4	14	23	37	397
CT‐12	1	17	2	14	100	7	550
CT‐14	1	24	3	7	24	7	233
CT‐18	36	101	4	56	23	37	402
CT‐19	1	22	3	7	72	7	439
CTB‐1	1	7	4	7	2	1	984
CTB‐2	1	24	3	7	24	7	233
CTB‐3	1	19	2	14	100	3	404
CTB‐4	1	16	3	7	9	3	23
CTB‐5	36	101	1	56	23	46	1419
CTB‐6	36	101	1	56	23	46	1419
CTB‐7	1	19	2	14	100	3	404
CTB‐8	1	46	12	76	60	9	708
CTB‐9	5	7	3	23	18	7	944
CTB‐10	5	89	3	23	18	4	400
CTB‐11	5	89	3	23	18	4	400
CTB‐12	1	24	3	7	48	7	936
CTB‐14	1	7	4	7	85	7	612
CTB‐18	1	24	3	7	48	7	936
CTB‐19	5	7	3	23	18	7	944
CTR‐2	1	19	4	14	100	3	1420
CTR‐8	1	19	4	14	100	3	1420
CTR‐19	1	7	4	91	52	4	844
CTR‐26	1	19	2	14	100	3	404

Abbreviations: DST, diploid sequence type; MLST, multilocus sequence typing.

Combining the allele numbers from all six loci resulted in the identification of 21 distinct DSTs from the 34 *C. tropicalis* isolates. Interestingly, 3 of the 21 genotypes (DSTs 1418, 1419, and 1420) were novel and different from those on MLST database. DSTs 1419 and 1420 were the most commonly detected (2 of 34 isolates), followed by DST 1418 (1 of 34 isolates; Table 2).

**Table 2:** MLST distribution of 34 *Candida tropicalis* clinical isolates.

*Candida tropicalis* MLST	No. of isolates (%)
404	4 (12.0)
233	3 (8.9)
944	3 (8.9)
400	2 (5.9)
612	2 (5.9)
708	2 (5.9)
936	2 (5.9)
1419	2 (5.9)
1420	2 (5.9)
23	1 (2.9)
201	1 (2.9)
247	1 (2.9)
397	1 (2.9)
402	1 (2.9)
439	1 (2.9)
550	1 (2.9)
679	1 (2.9)
685	1 (2.9)
844	1 (2.9)
984	1 (2.9)
1418	1 (2.9)
Total	34 (100.0)

Abbreviation: MLST, multilocus sequence typing.

Among the 21 genotypes, DST 404 was the most prevalent, observed in four isolates with an incidence rate of 12.0% each. Two genotypes, DSTs 233 and 944, were detected in three isolates, with an incidence rate of 8.9% each. Six genotypes (DSTs 400, 612, 708, 936, 1419, and 1420) were identified in two isolates, with an incidence rate of 5.9% each. The remaining 12 genotypes (DSTs 23, 201, 247, 397, 402, 439, 550, 679, 685, 844, 984, and 1418) were unique to single isolates (Table 2).

A total of 48 heterozygous sites were detected, with Y = C + T being the most frequent (23/48, 47.9%), followed by R = A + G (12/48, 25.0%), W = A + T and K = G + T (each 5/48, 10.4%), and M = A + C (3/48, 6.2%) (Table 3). Across the six gene fragments analyzed, 48 polymorphic sites were identified: 4 in ICL1, 12 in MDR1, 2 in SAPT2, 14 in SAPT4, 11 in XYR1, and 5 in ZWF1a. Among these, SAPT2 showed the highest typing efficiency, yielding 2.50 genotypes per polymorphic site, while SAPT4 had the lowest, with only 0.64 genotypes per polymorphism.

**Table 3:** Characteristics of six MLST housekeeping genes analyzed in this study.

Gene fragment	Heterozygosity						No. of heterozygotes	No. of polymorphic sites	No. of genotypes	Ratio of alleles to polymorphism
	Y	R	W	K	M	S				
*ICL1*	2	2	0	0	0	0	4	4	5	1.25
*MDR1*	4	5	2	1	0	0	12	12	12	1.00
*SAPT2*	0	1	0	1	0	0	2	2	5	2.50
*SAPT4*	6	4	1	2	1	0	14	14	9	0.64
*XYR1*	8	0	2	0	1	0	11	11	14	1.27
*ZWF1a*	3	0	0	1	1	0	5	5	8	1.60
Total	23	12	5	5	3	0	48	48	53	

### 3.3 Phylogenetic and Population Structure Analyses

**Figure 1:**
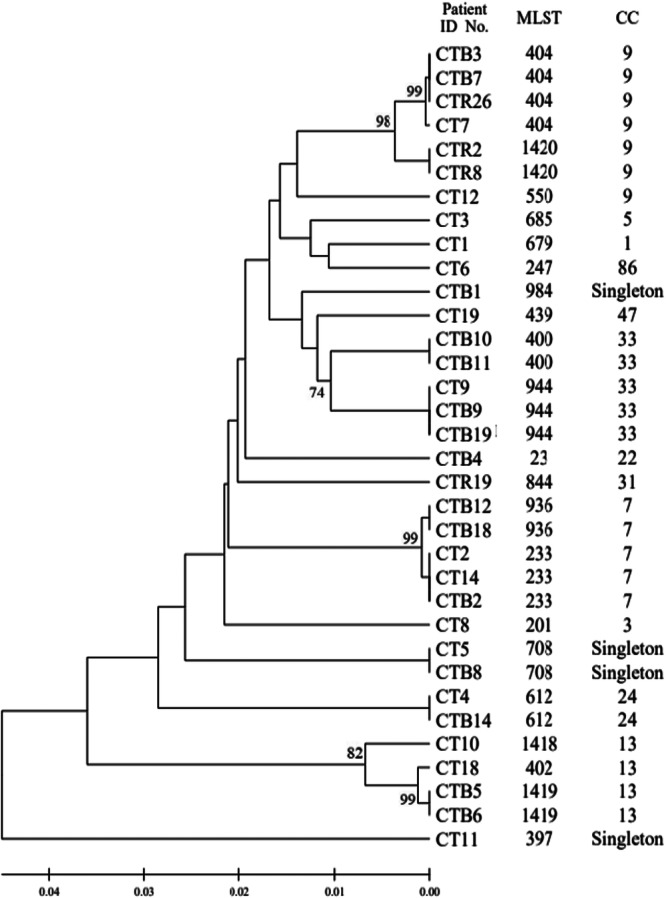
UPGMA dendrogram illustrating the phylogenetic relationships among 34 *Candida tropicalis* clinical isolates collected from Korea. Genetic distances were calculated using the *p*‐distance method, and bootstrap values greater than 70% are shown at the corresponding nodes.

**Figure 2:**
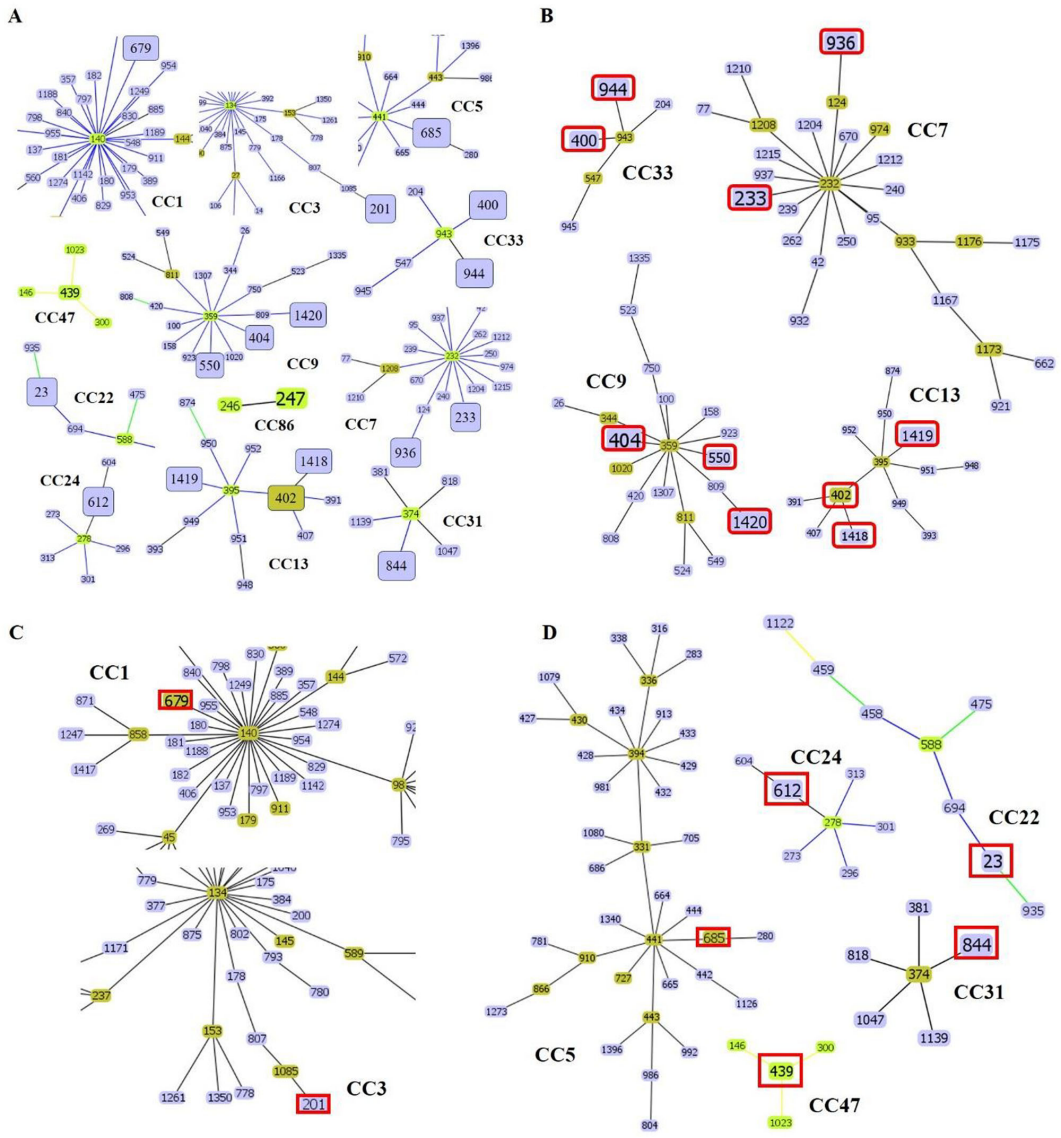
Population structure of *Candida tropicalis* clinical isolates. (A) goeBURST analysis illustrating a population snapshot of 21 validated diploid sequence types (DSTs). Clonal complexes identified in this study include CC7, CC9, CC13, and CC33 (B); CC1 and CC3 (C); and CC5, CC22, CC24, CC31, and CC47 (D), each containing DSTs found among the analyzed isolates.

To investigate the genetic relationships among the 21 DSTs of *C. tropicalis* isolates, phylogenetic analysis was performed using UPGMA with the MEGA 7 software (Figure 1). The eBURST program was used to identify the genotype clusters and compare them with the phylogenetic analysis results. Genetic relationships between the 34 Korean isolates used in this study and 1421 *C. tropicalis* strains on the MLST database until November 2022 were compared. GoeBURST analysis revealed that 18 DSTs were grouped into 11 CCs, whereas three DSTs were identified as singletons (Figures 1 and 2A). CC9 contained three DSTs (404, 550, and 1420), followed by CC33 with two DSTs (400 and 944), CC7 with two DSTs (233 and 936), cluster CC13 with three DSTs (402, 1418, and 1419), CC1 with DST 679, CC3 with DST 201, CC5 with DST 685, CC22 with DST 23, CC24 with DST 612, CC31 with DST 844, CC47 with DST 439, and CC86 with DST 247 (Figure 2B–D).

**Figure 3:**
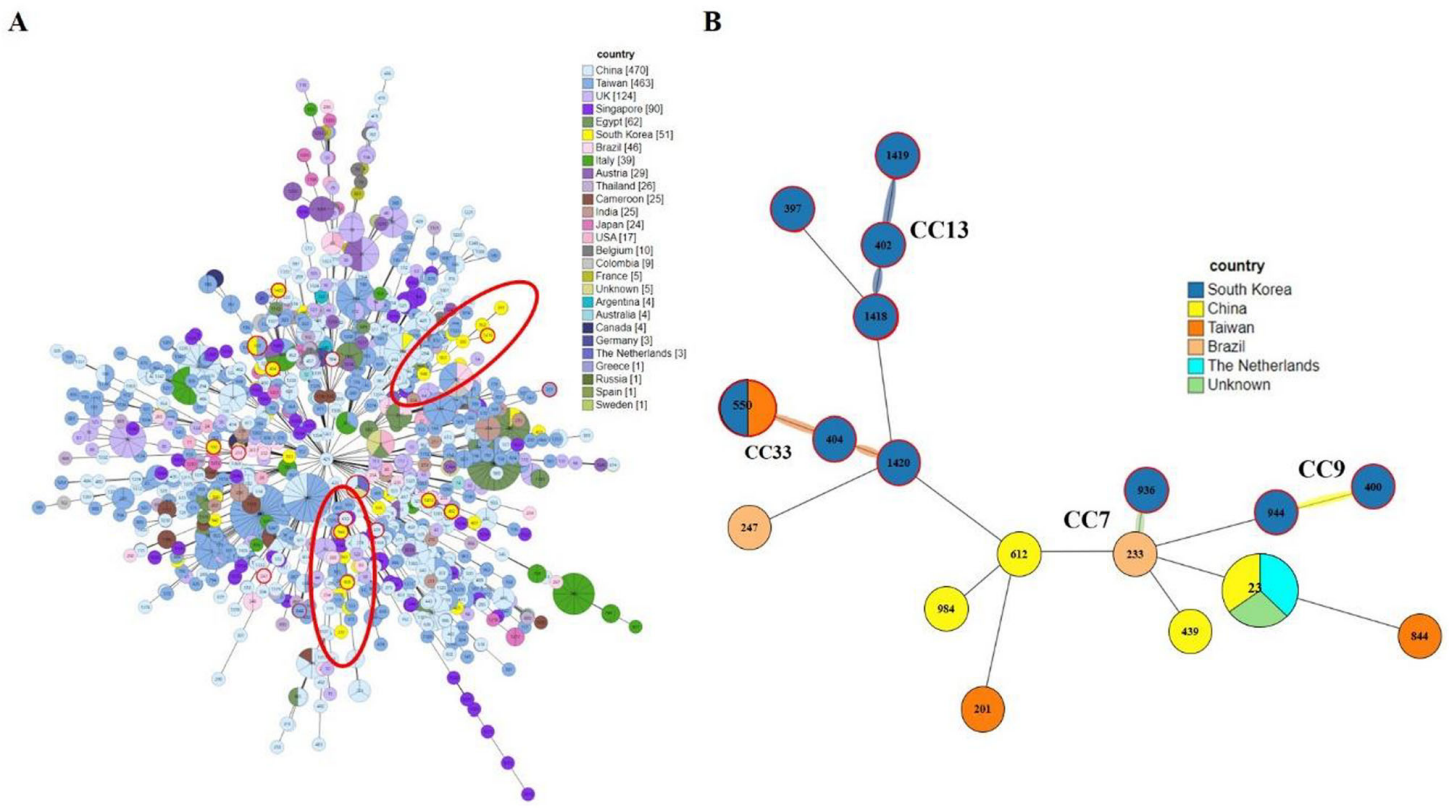
Minimum spanning tree (MST) representing *Candida tropicalis* clinical isolates. (A) Each circle's color corresponds to the country of origin, while the circle size reflects the number of isolates associated with a particular diploid sequence type (DST). (B) MST displaying Korean DSTs grouped into clonal complexes (CCs), including CC7, CC9, CC13, and CC33, with their global counterparts.

Comparison of the distribution of STs across different countries revealed that China had the highest number of DSTs, followed by Taiwan. In Korea, DST data were available for 51 isolates of *C. tropicalis* (Figure 3A). Among the CCs analyzed in this study, CC7, CC9, CC13, and CC33, which showed widespread distribution, were closely related to the Chinese, Taiwanese, and Brazilian lineages (Figure 3B).

To investigate the population structure of *Candida tropicalis* isolates, a PHI test for recombination was conducted using concatenated MLST sequences from all 34 isolates. The test yielded a *p*‐value of 0.0045, indicating statistically significant evidence of recombination among the analyzed loci. In addition, a NeighborNet phylogenetic network was constructed using SplitsTree4, which revealed multiple reticulate relationships among isolates, further supporting the presence of genetic recombination (Supporting Information S1: Figure S1).

## 4 Discussion

Recently, invasive fungi have become a major public health issue (Brown et al. 2012). According to the World Health Organization, fungal infections pose serious threats to human health, national economies, and health systems. *Candida* infections can be fatal in severely immunocompromised or debilitated individuals (Arastehfar, Daneshnia et al. 2020). Genetic analyses are crucial as the epidemiology of diseases caused by invasive species is influenced by many factors, such as geographical regions, patient characteristics, and localities (Scordino et al. 2018). Candidemia caused by *C. tropicalis* exhibits the highest mortality (Boonsilp et al. 2021; Yang et al. 2023). Despite its high mortality rate, recent global analysis of Korean MLST genotypes revealed gaps in the genetic information of various genotypes and their antifungal resistance, specimen types, and geographic locations.

*C. tropicalis*, similar to *C. albicans*, is diploid. In this study, MLST analysis, which is useful to examine the genetics of diploid yeast pathogens, was used (Wang, Li et al. 2020). Six loci were selected based on previous reports. *C. tropicalis* isolates can be easily distinguished with a high level of reproducibility using a specific set of six gene fragments (Magri et al. 2013). Among the six sequenced loci, *SAPT2* exhibited the highest, whereas SAPT4 exhibited the lowest typing efficiency. These findings differ from those reported in earlier studies. For instance, Tavanti et al. found that STAP4 had the highest typing efficiency, while XYR1 had the lowest (Tavanti et al. 2005). Conversely, Wang et al. observed that SAPT4 demonstrated the highest efficiency, detecting 1.54 genotypes per polymorphic site, whereas SAPT2 showed the lowest efficiency, identifying only 0.22 genotypes per polymorphism (Wang, Tang et al. 2020). These contrasting results may be due to variations in the sample processing methods, sample types, and characteristics of recruited study groups.

In this study, we identified 18 DSTs, along with three novel DSTs, from 34 *C. tropicalis* clinical isolates from Korea. Three DSTs (1418, 1419, and 1420) were newly discovered and not previously recorded on the MLST central database. DSTs 1419 and 1420 were the most common, observed in 2 of 34 isolates, whereas genotype DST 1418 was only observed in 1 of 34 isolates. The isolates containing DSTs 1418 and 1419 were obtained from patients with pneumonia.

GoeBURST analysis grouped 18 DSTs into 11 CCs and identified three DSTs as singletons. Notably, CC9 contained DSTs 404, 550, and 1420, CC33 contained DSTs 400 and 944, CC7 contained DSTs 233 and 936, CC13 contained DSTs 402, 1418, and 1419, and other CCs contained DSTs 679, 201, 685, 23, 612, 844, 439, and 247. We also compared the distribution of STs across different countries. Among the CCs analyzed in this study, CC7, CC9, CC13, and CC33 showed widespread distribution and were similar to the Chinese, Taiwanese, and Brazilian lineages. The overall genetic architecture of the *C. tropicalis* population is still not well defined, primarily because of the increasing diversity arising from the continual identification of various clinical and environmental isolates (Wang, Li et al. 2020; Wu et al. 2017; Scordino et al. 2018; Wang, Tang et al. 2020). This is consistent with our MLST data revealing that over 14% of the DSTs detected in this study were novel.

To assess the potential occurrence of genetic recombination within the *C. tropicalis* population, a PHI test was performed using concatenated MLST sequences from all 34 isolates (Bruen et al. 2006). The resulting *p*‐value of 0.0045 indicates statistically significant evidence of recombination among the analyzed loci. This finding challenges the assumption of a strictly clonal population structure and suggests that recombination events may contribute to the genetic diversity observed in the Korean clinical population. The reticulate patterns observed in the NeighborNet network further reinforce this interpretation (Huson and Bryant 2006). Together, these results highlight the complex evolutionary dynamics of *C. tropicalis*, a diploid yeast capable of both clonal expansion and recombination and emphasize the need for complementary phylogenetic methods when evaluating population structure.

In addition to genotypic diversity, phenotypic variability was also observed. For example, isolate CTR‐19, which was classified as DST844, showed elevated MIC to fluconazole. While DST844 or its associated clonal complex (CC31) has not previously been linked to azole resistance, this finding may represent a novel resistance phenotype. This highlights the importance of ongoing surveillance and further molecular characterization of emerging DSTs (Wang et al. 2023).

This study characterized the genotypic diversity of 34 *C. tropicalis* clinical isolates from Korea, identifying 18 DSTs, including three novel ones (DST1418, DST1419, and DST1420). Although these novel DSTs did not show distinct antifungal resistance profiles, our findings contribute to the expansion of the global MLST database and underscore the importance of continuous genotypic monitoring. To enhance the utility of MLST data, integration with high‐quality clinical and phenotypic metadata—including MIC values, specimen sources, and patient characteristics—is essential. Such a comprehensive approach, supported by advanced bioinformatic tools, will improve fungal genetic tracking, enhance the prediction of antifungal resistance trends, and support more effective infection control strategies across diverse clinical settings (Dougue et al. 2022).

This study is limited by the relatively small sample size and its restriction to three tertiary hospitals in Korea. Consequently, the identified DSTs may not fully represent the broader epidemiological or evolutionary characteristics of *C. tropicalis* in the region. Future studies should incorporate a larger and more geographically diverse set of clinical isolates to improve representativeness. Furthermore, while MLST remains a widely accepted and standardized method for studying diploid yeasts, it may not fully resolve fine‐scale genetic relationships or detect structural variations that influence phenotype (Zhang et al. 2004). In this context, whole‐genome sequencing (WGS) offers a more comprehensive approach by enabling genome‐wide detection of SNPs, copy number variations, tandem duplications, and resistance‐associated genes (Brassington et al. 2025; Smithgall et al. 2025; Collins et al. 2021; Keighley et al. 2022). A recent WGS‐based study by Fan et al. demonstrated that tandem gene duplications were responsible for azole resistance in a rapidly expanding *C. tropicalis* population (Fan et al. 2024). Therefore, integrating MLST with WGS and other high‐resolution methods in future studies will help refine genotypic classification and improve our understanding of the clinical and evolutionary significance of novel sequence types.

## Author Contributions

**Hye‐won Park:** formal analysis, methodology, writing – original draft. **Lia Kim:** formal analysis, methodology, writing – original draft. **Yoon‐Sung Choi:** investigation, methodology. **Jinyoung Bae:** investigation, methodology. **Min‐Ho Yeo:** investigation, methodology. **Eun Ju Lee:** investigation, methodology. **Jiyoung Lee:** investigation, methodology, writing – review and editing. **Kwangmin Park:** investigation, methodology. **Dong Geon Lee:** investigation, methodology. **Min Park:** supervision, writing – review and editing. **Sunghyun Kim:** conceptualization, supervision, funding acquisition, writing – review and editing. **Jungho Kim:** conceptualization, supervision, funding acquisition, writing – review and editing.

## Ethics Statement

The authors have nothing to report.

## Conflicts of Interest

The authors declare no conflicts of interest.

## Supporting information

Supporting Information revised KJsdf.

## Data Availability

The data that support the findings of this study are available from the corresponding author upon reasonable request.
