# Supplementary material for: Genotypic Analysis of Candida tropicalis Clinical Isolates From Korea via Multilocus Sequence Typing
Source: Microbiologyopen. 2025 Jun 29;14(4):e70024. doi: 10.1002/mbo3.70024 (PMC12206949; doi:10.1002/mbo3.70024)
Supplement: Supplementary file 1 — Supporting Information revised KJsdf. [file MBO3-14-e70024-s001.dot]

**Supporting Information**

**Genotypic analysis of *Candida tropicalis* clinical isolates from Korea via multilocus sequence typing**

**Running title: MLST analysis for *C. tropicalis* clinical isolates from Korea**

Hye-won Park^1,†^, Lia Kim^2,†^, Yoon-Sung Choi^3^, Jinyoung Bae^1^, Min-Ho Yeo^1^, Eun Ju Lee^1,4^, Jiyoung Lee^5^, KwangMin Park^1,6^, Dong Geon Lee^1,6^, Min Park^7^, Sunghyun Kim^1,6,*^, and Jungho Kim^1,6,*^

^1^Department of Clinical Laboratory Science, College of Health Sciences, Catholic University of Pusan, Busan 46252, Republic of Korea

^2^Department of Biomedical Laboratory Science, College of Health Sciences, Yonsei University, Wonju 26493, Republic of Korea

3Department of Thoracic and Cardiovascular Surgery, Inje University Haeundae Paik Hospital, Inje University College of Medicine, Busan 48108, Republic of Korea

^4^Korea Mycobacterium Resource Center (KMRC), Department of Research and Development, The Korean Institute of Tuberculosis, Osong 28158, Republic of Korea

^5^Department of Research & Development, DreamDX Inc., C001, 57, Oryundae-ro, Geumjeong-gu, Busan, 46252, Republic of Korea

^6^Next-Generation Industrial Field-Based Specialist Program for Molecular Diagnostics, Brain Busan 21 Plus Project, Graduate School, Catholic University of Pusan

^7^Department of Biomedical Laboratory Science, Masan University, Changwon, 51217, Republic of Korea

^†^These authors have contributed equally to this work.

**^*^Corresponding authors**:

**Sunghyun Kim, PhD**

Mailing address: Department of Biomedical Laboratory Science, College of Health Sciences, Catholic University of Pusan, Busan 46252, Republic of Korea

Tel: +82-51-510-0660

Fax: +82-51-510-0568

E-mail: shkim0423@cup.ac.kr

**Jungho Kim, PhD**

Mailing address: Department of Biomedical Laboratory Science, College of Health Sciences, Catholic University of Pusan, Busan 46252, Republic of Korea

Tel: +82-51-510-0660

Fax: +82-51-510-0568

E-mail: [jutosa70@cup.ac.kr](mailto:jutosa70@cup.ac.kr)


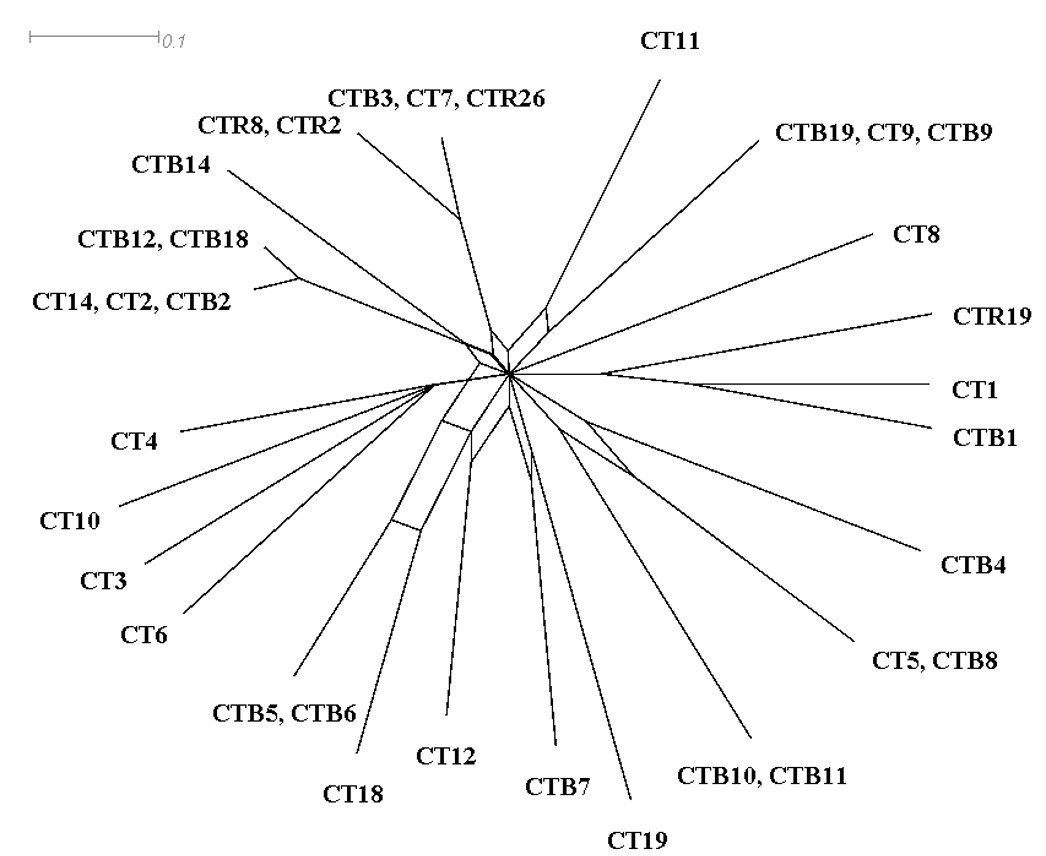


**FIGURE S1.** NeighborNet phylogenetic network constructed from concatenated MLST sequences of 34 *Candida tropicalis* clinical isolates using SplitsTree4. The network reveals reticulate relationships among several isolates, suggesting potential genetic recombination. The PHI test for recombination yielded a *p*-value of 0.0045, indicating statistically significant evidence of recombination among the loci analyzed.

**Supplementary Tables**

**Table S1.** Clinical information of *Candida tropicalis* isolates analyzed in this study.

| Sample | Gender | Age | Specimen type | Diagnosis |
| --- | --- | --- | --- | --- |
| CT-1 | M | 66 | Blood | Tonsillar cancer |
| CT-2 | F | 32 | Blood | Sepsis |
| CT-3 | F | 60 | Blood | Malignant neoplasm of the stomach |
| CT-4 | M | 78 | Blood | Alcoholic cirrhosis of the liver |
| CT-5 | F | 76 | Blood | Gonarthrosis |
| CT-6 | M | 79 | Blood | Pneumonia |
| CT-7 | M | 56 | Blood | T-cell prolymphocytic leukemia |
| CT-8 | F | 82 | Blood | Thoracic aortic aneurysm |
| CT-9 | M | 77 | Blood | Pneumonia |
| CT-10 | F | 75 | Blood | Pneumonia |
| CT-11 | M | 63 | Blood | Infective neoplasm of the prostate |
| CT-12 | M | 68 | Blood | Malignant neoplasm of the prostate |
| CT-14 | M | 41 | Blood | Myeloid leukemia |
| CT-18 | M | 98 | Blood | Non-small cell lung cancer |
| CT-19 | M | 65 | Blood | Malignant neoplasm of the bronchi and lungs |
| CTB-1 | M | 80 | Ascitic fluid | Malignant neoplasm of the pyloric antrum |
| CTB-2 | F | 85 | Ascitic fluid | Hypertension |
| CTB-3 | M | 62 | Bile | Rupture of bile duct |
| CTB-4 | F | 82 | Closed pus | Retroperitoneal abscess |
| CTB-5 | M | 94 | Closed pus | Pneumonia |
| CTB-6 | M | 62 | Closed pus | Other secondary gonarthrosis, bilateral |
| CTB-7 | F | 74 | Other | Malignant neoplasm of the duodenum |
| CTB-8 | F | 70 | Other | Cardiac arrest |
| CTB-9 | F | 71 | Other | Other endophthalmitis |
| CTB-10 | F | 67 | Other | Malignant neoplasm of the body of the stomach |
| CTB-11 | F | 72 | Blood | Non-Hodgkin lymphoma |
| CTB-12 | M | 68 | Blood | Malignant neoplasm of the descending colon |
| CTB-14 | M | 49 | Blood | End-stage kidney disease on dialysis |
| CTB-18 | M | 40 | Blood | Myeloid leukemia |
| CTB-19 | M | 68 | Blood | Pneumonia |
| CTR-2 | M | 66 | Blood | Fungal endocarditis |
| CTR-8 | M | 72 | Blood | Lung cancer, epiglottic cancer |
| CTR-19 | M | 76 | Blood | Coronary artery obstructive disease |
| CTR-26 | M | 54 | Blood | Rectal cancer |

**Table S2. Antifungal susceptibility testing results of 34 *C. tropicalis* clinical isolates**

| Sample | AMB | | CAS | | FLUCO | | FLUCY | | VOR | |
| --- | --- | --- | --- | --- | --- | --- | --- | --- | --- | --- |
|  | *MIC | S/I/R | MIC | S/I/R | MIC | S/I/R | MIC | S/I/R | MIC | S/I/R |
| CT-1 | ≤0.25 | S | ≤0.25 | S | ≤1 | S | ≤1 | S | ≤0.12 | S |
| CT-2 | ≤0.25 | S | ≤0.25 | S | ≤1 | S | ≤1 | S | ≤0.12 | S |
| CT-3 | 0.50 | S | ≤0.25 | S | ≤1 | S | ≤1 | S | ≤0.12 | S |
| CT-4 | 0.50 | S | ≤0.25 | S | ≤1 | S | ≤1 | S | ≤0.12 | S |
| CT-5 | ≤0.25 | S | ≤0.25 | S | ≤1 | S | ≤1 | S | ≤0.12 | S |
| CT-6 | 0.50 | S | ≤0.25 | S | ≤1 | S | ≤1 | S | ≤0.12 | S |
| CT-7 | 1.00 | S | ≤0.25 | S | ≤1 | S | ≤1 | S | ≤0.12 | S |
| CT-8 | 0.50 | S | ≤0.25 | S | ≤1 | S | ≤1 | S | ≤0.12 | S |
| CT-9 | 0.50 | S | ≤0.25 | S | ≤1 | S | ≤1 | S | ≤0.12 | S |
| CT-10 | 0.50 | S | ≤0.25 | S | ≤1 | S | ≤1 | S | ≤0.12 | S |
| CT-11 | 0.50 | S | ≤0.25 | S | ≤1 | S | ≤1 | S | ≤0.12 | S |
| CT-12 | 0.50 | S | ≤0.25 | S | ≤1 | S | ≤1 | S | ≤0.12 | S |
| CT-14 | ≤0.25 | S | ≤0.25 | S | ≤1 | S | ≤1 | S | ≤0.12 | S |
| CT-18 | 0.50 | S | ≤0.25 | S | ≤1 | S | ≤1 | S | ≤0.12 | S |
| CT-19 | 0.50 | S | ≤0.25 | S | ≤1 | S | ≤1 | S | ≤0.12 | S |
| CTB-1 | ≤0.25 | S | ≤0.25 | S | ≤1 | S | ≤1 | S | ≤0.12 | S |
| CTB-2 | ≤0.25 | S | ≤0.25 | S | ≤1 | S | ≤1 | S | ≤0.12 | S |
| CTB-3 | ≤0.25 | S | ≤0.25 | S | ≤1 | S | ≤1 | S | ≤0.12 | S |
| CTB-4 | ≤0.25 | S | ≤0.25 | S | ≤1 | S | ≤1 | S | ≤0.12 | S |
| CTB-5 | ≤0.25 | S | ≤0.25 | S | ≤1 | S | ≤1 | S | ≤0.12 | S |
| CTB-6 | ≤0.25 | S | ≤0.25 | S | ≤1 | S | ≤1 | S | ≤0.12 | S |
| CTB-7 | None | S | ≤0.25 | S | ≤1 | S | ≤1 | S | ≤0.12 | S |
| CTB-8 | None | S | ≤0.25 | S | ≤1 | S | ≤1 | S | ≤0.12 | S |
| CTB-9 | ≤0.25 | S | ≤0.25 | S | ≤1 | S | ≤1 | S | ≤0.12 | S |
| CTB-10 | ≤0.25 | S | ≤0.25 | S | ≤1 | S | ≤1 | S | ≤0.12 | S |
| CTB-11 | None | S | ≤0.25 | S | ≤1 | S | ≤1 | S | ≤0.12 | S |
| CTB-12 | None | S | ≤0.25 | S | ≤1 | S | ≤1 | S | ≤0.12 | S |
| CTB-14 | ≤0.25 | S | ≤0.25 | S | ≤1 | S | ≤1 | S | ≤0.12 | S |
| CTB-18 | ≤0.25 | S | ≤0.25 | S | ≤1 | S | ≤1 | S | ≤0.12 | S |
| CTB-19 | ≤0.25 | S | ≤0.25 | S | ≤1 | S | ≤1 | S | ≤0.12 | S |
| CTR-2 | ≤0.25 | S | ≤0.25 | S | ≤1 | S | ≤1 | S | ≤0.12 | S |
| CTR-8 | ≤0.25 | S | ≤0.25 | S | ≤1 | S | ≤1 | S | ≤0.12 | S |
| CTR-19 | ≤0.25 | S | ≤0.25 | S | 4 | I | 64 | R | ≤0.12 | S |
| CTR-26 | ≤0.25 | S | ≤0.25 | S | ≤1 | S | ≤1 | S | ≤0.12 | S |

AMB, amphotericin; CAS, caspofungin; FLUCO, fluconazole; FLUCY, 5-flucytosin; VOR, voriconazole; R, resistant; I, intermediate; S, susceptible; *MIC unit, mg/L

**Table S3.** Primers used to identify the *C. tropicalis* isolates tested in this study.

| Primer | Primer sequence (5′–3′) | Amplicon size (bp) | Reference |
| --- | --- | --- | --- |
| *ITS1-F* | TCCGTAGGTGAACCTGCGG | 560 | 14 |
| *ITS4-R* | TCCTCCGCTTATTGATATGC |

ITS, Internal transcribed spacer

**Table S4.** List of gene fragments and primers used for *C. tropicalis* multilocus sequence typing (MSLT).

| Gene fragment | Gene product | Primer sequence (5′–3′) | | Amplicon size (bp) | Reference |
| --- | --- | --- | --- | --- | --- |
| *ICL1* | Isocitrate lyase | F | CAACAGATTGGTTGCCATCAGAGC | 737 | 20 |
|  |  | R | CGAAGTCATCAACAGCCAAAGCAG |  |  |
| *MDR1* | Multidrug resistance protein | F | TGTTGGCATTCACCCTTCCT | 663 |  |
|  |  | R | TGGAGCACCAAACAATGGGA |  |  |
| *SAPT2* | Secreted aspartic protease 2 | F | CAACGATCGTGGTGCTG | 658 |  |
|  |  | R | CACTGGTAGCTGAAGGAG |  |  |
| *SAPT4* | Secreted aspartic protease 4 | F | TGCTTCTCCTACAACTTCACCTCC | 483 |  |
|  |  | R | ATTCCCATGACTCCCTGAGCAACA |  |  |
| *XYR1* | D-xylose reductase | F | AGTTGGTTTCGGATGTTG | 479 |  |
|  |  | R | TCGTAAATCAAAGCACCAGT |  |  |
| *ZWF1a* | Putative glucose-6-phosphate dehydrogenase | F | GGTGCTTCAGGAGATTTAGC | 647 |  |
|  |  | R | ACCTTCAGTACCAAAAGCTTC |  |  |

F, forward; R, reverse
